# Green synthesis, characterisation and biological evaluation of plant‐based silver nanoparticles using *Quercus semecarpifolia* Smith aqueous leaf extract

**DOI:** 10.1049/iet-nbt.2018.5063

**Published:** 2018-10-24

**Authors:** Aishma Khattak, Bashir Ahmad, Abdur Rauf, Sami Bawazeer, Umar Farooq, Jawad Ali, Seema Patel, Eman Ramadan El‐Sharkawy, Rabia Ikram, Huang Linfang

**Affiliations:** ^1^ Centre of Biotechnology and Microbiology University of Peshawar Pakistan; ^2^ Department of Chemistry University of Swabi Anbar Swabi KP Pakistan; ^3^ Department of EMS Paramedic College of Public Health and Health Informatics, Umm Al‐Qura University Makkah Saudi Arabia; ^4^ Department of Chemistry COMSATS Institute of Information Technology Abbottabad 22060 Pakistan; ^5^ Bioinformatics and Medical Informatics Research Center, San Diego State University San Diego 92182 USA; ^6^ Department of Chemistry Faculty of Science Northern Borders University Saudi Arabia; ^7^ Department of Chemistry Center for Natural Products and Drug Research (CENAR), University of Malaysia Kuala Lumpur Malaysia; ^8^ Institute of Medicinal Plant Development (IMPLAD) Peking Union Medical College Chinese Academy of Medical Sciences (CAMS) No.151, Malianwa North Road, HaiDian District Beijing 100193 People's Republic of China

**Keywords:** scanning electron microscopy, toxicology, visible spectra, particle size, nanofabrication, nanomedicine, transmission electron microscopy, silver, ultraviolet spectra, differential thermal analysis, nanoparticles, X‐ray diffraction, botany, biochemistry, cellular biophysics, green synthesis, biological evaluation, plant‐based silver nanoparticles, reliable methods, metallic nanoparticles, eco‐friendly cost‐effective protocol, silver NPs, ultraviolet–visible spectroscopic analysis, highest absorbance peak, particle size, structure, transmission electron microscopy analysis, TEM imaging, crystalline nature, X‐ray powder diffraction patterns, differential thermal analysis, pharmacological evaluation, aqueous extracts, good cytotoxic activity, significant antioxidant activity, AgNPs exhibited good phytotoxic potential, bio‐inspired synthesis, Quercus semecarpifolia Smith aqueous leaf extract, scanning electron microscopy, thermogravimetry, crude methanolic, n‐hexane, chloroform, ethyl acetate, phytotoxic potential, haemagglutination activity, size 20.0 nm to 50.0 nm, wavelength 430.0 nm, temperature 100 degC to 1000 degC, Ag

## Abstract

The development of reliable and green methods for the fabrication of metallic nanoparticles (NPs) has many advantages in the field of nanotechnology. In this direction, the present work describes an eco‐friendly and cost‐effective protocol for the production of silver NPs (AgNPs) using an aqueous extract of *Quercus semecarpifolia* leaves. Different techniques were carried out for the characterisation of the synthesised AgNPs. The ultraviolet–visible spectroscopic analysis showed the highest absorbance peak at 430 nm. The particle size and structure were confirmed by scanning electron microscopy as well as transmission electron microscopy (TEM) analysis. From TEM imaging, it was revealed that the formed particles were spherical with an average size of 20–50 nm. The crystalline nature of the NPs was determined by X‐ray powder diffraction patterns. Thermogravimetry and differential thermal analysis were also evaluated by a temperature increment from 100 to 1000°C. Bio‐inspired synthesis of AgNPs was performed for their pharmacological evaluation in relation to the activities of the crude methanolic, *n* ‐hexane, chloroform, ethyl acetate, and aqueous extracts. Good cytotoxic activity was exhibited by the green‐synthesised AgNPs (77%). Furthermore, the AgNPs were found to exhibit significant antioxidant activity at 300 μg/ml (82%). The AgNPs also exhibited good phytotoxic potential (75%).

## 1 Introduction

Nanotechnology has emerged as an interdisciplinary science with applications in biomedical sciences, pharmacology, food processing [1, 2, 3], cosmetics formulation, optics, electronics, and chemical industries. Research and progress in this field are growing rapidly throughout the world [2].

Silver nanoparticles (AgNPs) are of much importance because of their distinctive nature and exclusive characteristics such as catalytic, optical, electrical and most importantly, antimicrobial properties [4, 5, 6]. These attributes render them unique among all metallic nanoparticles (NPs) [7]. Owing to these properties, they have been successfully implicated in the biomedical field for diagnostics, pharmacology, molecular imaging and drug delivery [8]. Also, they are being added to topical creams, wound dressings, antiseptic sprays and textile industry [9, 10].

In view of their wide‐range uses, the fabrication of AgNPs of distinctive structures and sizes is a much‐pursued field of study [11, 12]. Various physical and chemical methods are used for the synthesis of AgNPs, but most of these methods are expensive and require the usage of toxic solvents [13]. Recently, green synthesis of environment‐friendly metal NPs processes using algal, fungal, and plant extracts have received much attention [14, 15]. This method exploits the phytochemicals for reduction of metal ions to NPs [16]. Phytochemicals such as terpenoids, phenols, tannins, alkaloids, flavonoids, quinines *etc*. act as reducing agents in the synthesis of NPs. These green methods have several advantages such as the easy availability, low cost, and formation of crystalline NPs ranging in size between 1 and 100 nm. The green synthesis potential of different plants varies due to their unique phytochemical profile. *Quercus semecarpifolia* Smith a large gregarious tree, commonly known as brown oak, was selected for the present study. *Quercus* is an important genus of family Fagaceae, and it has about 45 species, among which *Q. semicarpifolia* is highly distributed in the temperate regions of Pakistan. Different parts of oak trees have therapeutic properties, and they are extensively used in traditional medicine as an analgesic, antipyretic and anti‐inflammatory medications [16]. The preliminary phytochemical investigation of *Q. semicarpifolia* has revealed that it has a high amount of flavonoid, phenolic and tannin components with strong antioxidant potential. With such a rich biological potency, this plant might be a suitable candidate for the fabrication of metal NPs. Thus, the aim of the present study was to evaluate the simple, rapid and reliable method of AgNP production using *Q. semicarpifolia* leaves extract and evaluation of their pharmacological activities [17].

## 2 Experimental

### 2.1 Plant collection

The plant material of *Q. semecarpifolia* was collected from District Mansehra, Khyber Pakhtunkhwa (KPK), Pakistan. The identification of plant was done by Dr Habib Ahmad, Vice‐Chancellor, Islamia College University Peshawar, Pakistan.

### 2.2 Extraction and fractionation

The shade‐dried plant was pulverised into powder form and kept at room temperature. Analytical grade methanol was used twice for soaking the plant material and kept at room temperature for 15 days. Stirring of the soaked plant material was done from time to time. The filtrate was concentrated using a rotary evaporator under vacuum at 40°C, to obtain a crude methanolic extract. For the fractionation of the crude methanolic extract, different solvents (*n* ‐hexane, chloroform, and ethyl acetate) were utilised.

### 2.3 Green biogenic synthesis of AgNPs

Green AgNPs were synthesised by using the aqueous leaf extract of *Q. semecarpifolia*. In 500 ml of distilled water, 25 g of powdered leaves were mixed and boiled for 30 min in order to make the aqueous leaf extract. The extract obtained was filtered using Whatman No.1 filter paper to obtain a clear solution. The aqueous extract was centrifuged twice at 10,000 rpm for 15 min to remove cell debris. The resultant extract was further filtered through a 0.2 μm filter paper and subsequently used for the fabrication of AgNPs*.* In 90 ml of AgNO_3_ (1.0 mM) solution, 10 ml of aqueous leaf extract was added, followed by heating in shaking water bath at 75°C for 1 h. The reduction of Ag^+^ ions to Ag^0^ NPs was observed by the change in colour of the solution from light yellow to dark brownish. The mixture was centrifuged at 10,000 rpm for 15 min at 4°C and re‐dispersed in distilled water to remove any unbound phytochemicals. Finally, the solution was concentrated using a rotary evaporator and dried.

### 2.4 Characterisation of bio‐inspired AgNPs

#### 2.4.1 Ultraviolet–visible (UV–Vis) spectral study

UV–Vis spectroscopy is a simple and effective technique to confirm the formation of NPs [18]. A double beam spectrophotometer (Shimadzu UV‐1650PC spectrophotometer) was used for UV–Vis analysis. Precisely, 4 ml of the diluted AgNPs sample was placed in a cuvette and inserted into the UV–Vis spectrophotometer to obtain the UV–Vis spectrum of the sample in the wavelength range of 300–800 nm.

#### 2.4.2 X‐ray diffraction (XRD) dimension

The crystallographic structure of purified AgNPs was detected using an XRD spectrum [19]. A thin layer of AgNPs was applied on a carbon‐coated copper grid and examined by using an X‐ray diffractometer (X'Pert‐Pro) operated with Cu Kα radiation and a current of 30 mA. The scanning was done in the region of 2*θ* from 10° to 80°.

#### 2.4.3 Scanning electron microscopy (SEM) analysis

The SEM investigation was done using a (Hitachi S‐4200) SEM machine. Thin layers of the AgNPs were prepared and loaded on the copper grid by dipping a small quantity of the test sample on the lattice. The blotting sheet was used to eliminate the extra solution. Using a mercury lamp, the sample was dried for 5 min and the images of NPs were taken.

#### 2.4.4 Transmission electron microscopy (TEM)

TEM was conducted utilising a transmission electron microscope (Techni‐G2‐300kV). Using distilled water, AgNPs were loaded on the carbon‐coated copper grid and dried. The TEM analysis predominantly confirmed the size and morphology of the AgNPs [20].

#### 2.4.5 Energy dispersive X‐ray (EDX) spectrum

EDX spectroscopy (EDX‐JEOL – 2300) was used to confirm the elemental configuration of AgNPs. The EDX technique predominantly detected the radiation released from the sample during the process of bombardment by a stream of electrons [21].

#### 2.4.6 Thermo gravimetric and differential thermal analysis (TG–DTA)

The thermogravimetry analysis (TGA) and DTA studies were performed using a thermal analysis system (STA7200, Hitachi). The powder form of AgNPs was used for the investigation. Two glass aluminium containers, one containing the sample and another containing reference, tied together but not touching each other, were subjected to heating from 100 to 1000°C using a persistent stream of nitrogen gas. During the investigation, the level of gas was checked frequently [22].

### 2.5 In vitro bioassay screening of AgNPs

#### 2.5.1 Antioxidant activity

The antioxidant activity of synthesised AgNPs and crude extracts was evaluated by their ability to eliminate the free radicals using 1,1‐diphenyl‐2‐8 picrylhydrazyl (DPPH) as per the reported procedure [23]. About 0.01 g of synthesised AgNPs and each extract was dissolved in 1 ml of methanol for making a stock solution. Furthermore, the test samples were diluted to make three concentrations (100, 200, 300 μg/ml). 1 ml from each concentration was mixed with 0.2 ml of freshly prepared DPPH solution. For positive and negative control, ascorbic acid (20–250 μg/ml) and solution without DPPH, respectively, were used. All of the samples were placed in the dark for 30 min followed by absorbance measurement at 517 nm.

#### 2.5.2 Brine shrimp lethality bioassay

The cytotoxic potential of AgNPs and crude extracts was evaluated by Brine shrimp (*Artemia salina*) lethality assay [24]. A good medium for hatching of Brine shrimp eggs is sea water. Artificial sea water was prepared by dissolving 38 g NaCl in 0.1 l of distilled water. Eggs (100 mg) were introduced to this solution and allowed to hatch and mature at room temperature for two days. The nauplii were collected using a Pasteur pipette. Further stock solution (20 mg/ml) of test samples was prepared in methanol (2 ml) and diluted to get final concentrations of 1000, 100 and 10 μg/ml and were transferred to sterile flasks. The flasks were kept in laminar flow hood for some time in order to vaporise the solvent. Ten *A. salina* naupli with 0.1 ml of sea water was transferred to each flask and the final volume of each flask was adjusted to 5 ml with sea water. The flasks were incubated at 28°C for 24 h. Methanol and etoposide drug served as negative and positive control, respectively. After 24 h, using magnifying glass shrimp lethality was estimated and LC_50_ was determined.

#### 2.5.3 Phytotoxic activity

The phytotoxic activity of synthesised AgNPs and crude extracts was carried out using the *Lemna minor* bioassay protocol as reported earlier [25]. According to the protocol, firstly E‐medium was prepared in distilled water by mixing various constituents. The stock solution was prepared by dissolving test samples (20 mg) in methanol (1.5 ml). From this solution 10, 100 and 1000 µg/ml were transferred to sterile Petri plates and kept for some time until all the solvent evaporated. E‐medium (20 ml) along with 16 healthy *L. minor* plants were added to the Petri plates. Incubation of the plates was carried out at 28°C in a growth chamber for one week. For negative and positive control, E‐medium without plant extract and paraquat (0.015 µg/ml) were used, respectively. A number of fronds were counted on day seven and results were recorded.

#### 2.5.4 Haemagglutination activity

Certain plants possess haemagglutinins (phytolectins) which could provide a cheaper source as blood‐typing reagents. The haemagglutination assay of AgNPs and crude extracts was determined as per the reported procedure [23]. The haemagglutination activity was tested against human erythrocytes. Fresh blood was taken and centrifuged at 3000 rpm to obtain erythrocytes while the supernatant was discarded. Phosphate buffer (pH 7.2) was used for making a blood sample (2%). Stock solution (0.1 mg/ml of dimethylsulphoxide) was prepared in phosphate buffer from which different dilutions (1:2, 1:4, 1:8 and 1:16) were prepared. A total volume of 1 ml of each dilution of the test sample was mixed with 1 ml of red blood cells (RBCs) suspension. It was followed by incubation for 30–40 min at room temperature and was observed for any agglutination.

## 3 Results and discussion

### 3.1 Plant‐mediated synthesis of AgNPs

**Fig. 1:**
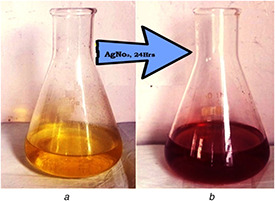
Visual observation of ***(a)*** Solution before bio‐reduction, ***(b)*** After bio‐reduction

According to the previous studies, it was reported that AgNPs possess dark brownish colour. The colour of *Q. semecarpifolia* leaf extract was light yellow before treating with silver nitrate (AgNO_3_) but when AgNO_3_ was added, the colour of the solution changed to dark brownish (Fig. 1), specifying the production of AgNPs*.* This is because of reducing the power of biomolecules present in the extract.

### 3.2 Characterisation of AgNPs

#### 3.2.1 UV–Vis spectroscopy

**Fig. 2:**
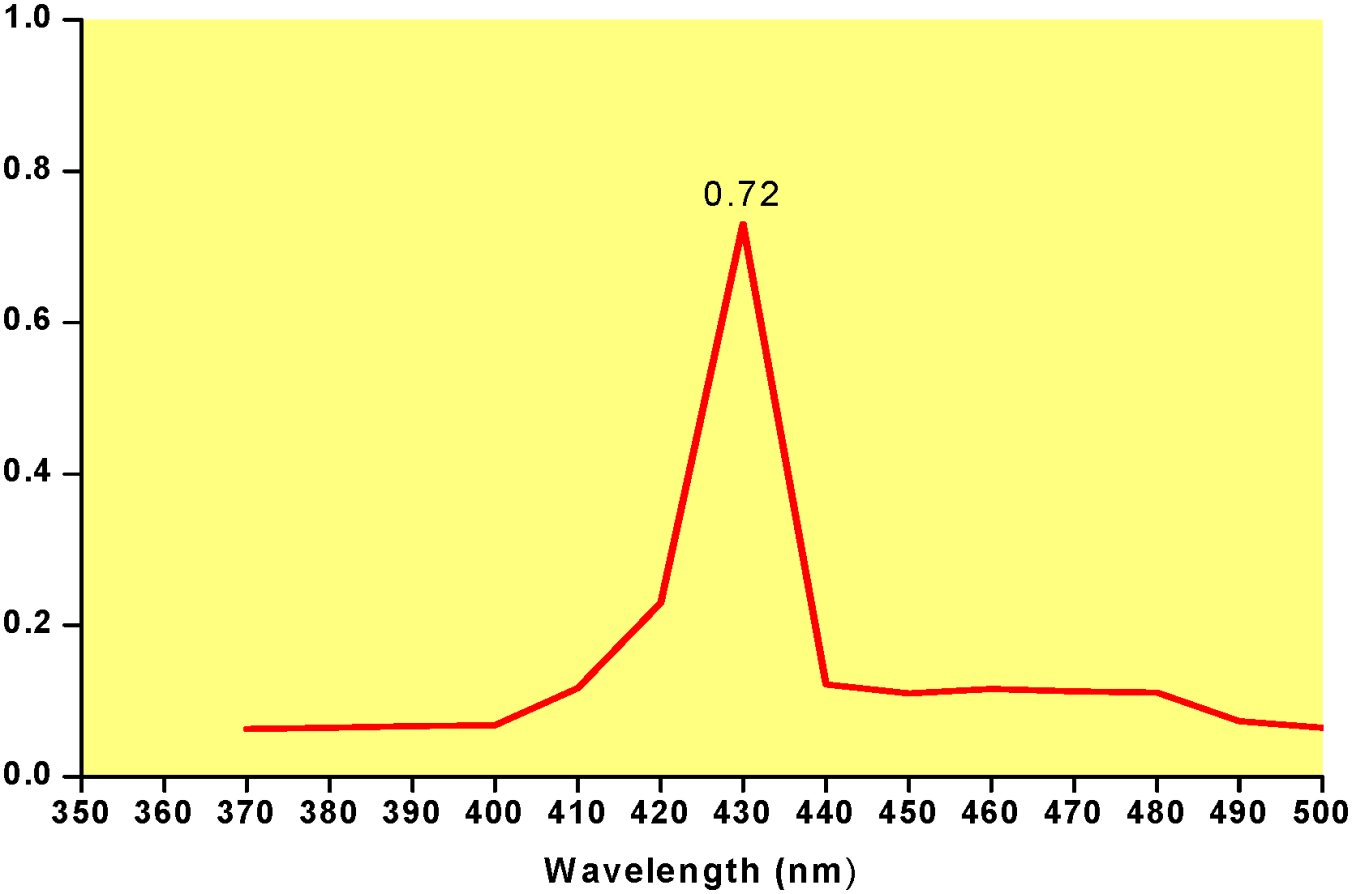
Graphical representation of UV–Vis spectroscopic assessment of green synthesised AgNPs from Q. semecarpifolia

Distinctive optical properties are exhibited by noble metals due to the property of surface plasmon resonance. The UV–Vis spectroscopy is generally used to detect the formation of AgNPs by measuring surface plasmon resonance peaks. On addition of the leaf extract of *Q. semecarpifolia* to the silver nitrate solution, the solution changes from light yellow to reddish brown. Due to the reduction of Ag^+^ to Ag^0^ by using the active phytochemicals present in the aqueous leaves extract, the colour of the solution was changed [26]. These phytochemicals act as both reducing and capping agents for the synthesis of NPs. Previously, it is reported that the diverse groups of biomolecules present in the extract are responsible for the synthesis of symmetrical NPs [27]. Fig. 2 shows the highest absorbance for AgNPs and was confirmed at around *λ*  = 430 nm. Biogenic AgNPs were stable for about 3 months.

#### 3.2.2 XRD measurements

**Fig. 3:**
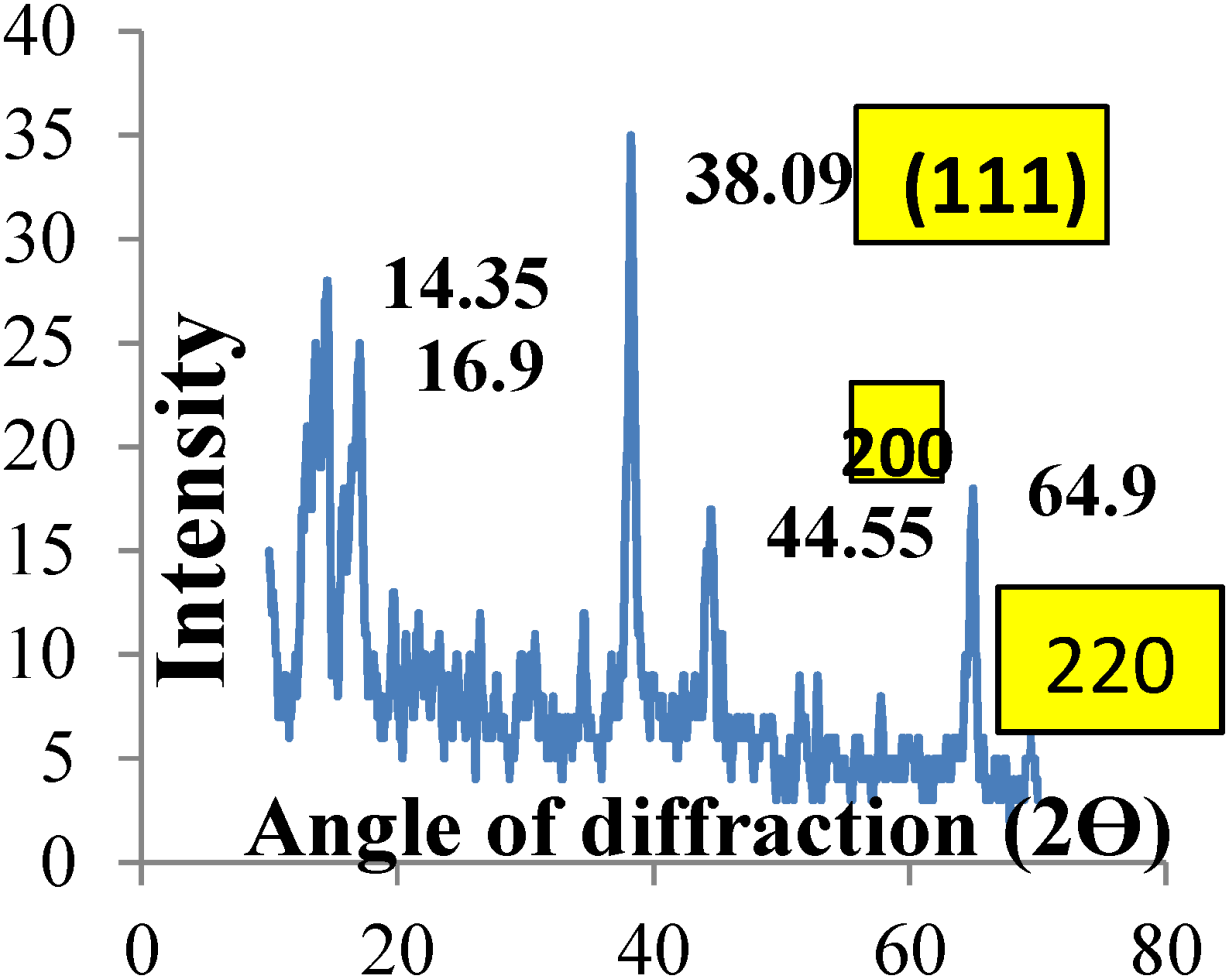
Graphical representation of XRD values of Q. semecarpifolia derived AgNPs

The XRD pattern is generally used for the confirmation of the crystalline nature of AgNPs. In the current study, an XRD investigation was performed to determine the crystal size of the samples (Fig. 3). From the XRD results, different peaks were recorded for the samples and according to the Debye–Scherrer equation, the size of the crystals was determined to be 8.5 Å for the synthesised AgNPs. The intensities of diffraction were recorded ∼64.9°, 44.55°, and 38.09° and were consigned to reflections from the 2*θ* region, corresponding to (220), (200) and (111) planes of silver, respectively. It is confirmed that they can be presented as face‐centred‐cubic structures of silver. Other peaks were also observed at 14.35° and 16.9°. They are previously reported in which the XRD pattern incorporated in the complete spectrum of 2*θ* ranging from 10° to 80°. These peaks were due to the biomolecules present in the plant extract and responsible for the fabrication of AgNPs [28].

#### 3.2.3 Scanning electron microscopy

**Fig. 4:**
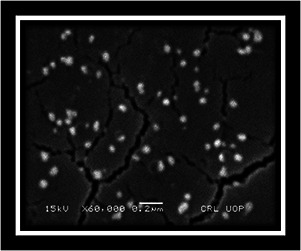
SEM micrograph of Q. semecarpifolia derived AgNPs

The structure and morphology of synthesised AgNPs from the *Q. semecarpifolia* leaf extract were observed by SEM. The images obtained from the SEM investigation clearly indicated that the morphology of AgNPs was spherical with an average size of 20–50 nm and well distributed without aggregation (Fig. 4)

Different capping and reducing agents in the leaf extract are responsible for the variation in size as reported earlier [29].

#### 3.2.4 Transmission electron microscopy

**Fig. 5:**
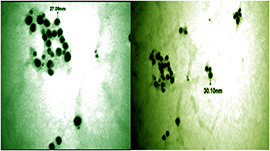
TEM micrograph of Q. semecarpifolia derived AgNPs ***(a)*** At 100 nm magnification, ***(b)*** At 200 nm magnification

The TEM is mostly used to confirm the size and morphology of bio‐inspired AgNPs. Fig. 5 shows the TEM image of biosynthesised AgNPs using *Q. semecarpifolia* leaf extract which predominates with uniform shape (spherical) morphologies ranging from 20 to 50 nm with an average size of 28.40 nm. Most of the AgNPs have spherical morphology with smooth edges and have ability to penetrate across the membrane [30, 31]. These structures were the same as those of the bio‐inspired AgNPs synthesised from the leaves of *Q. incana* and *Q. brantii* which was due to the resemblance in the biomolecules present in both plant species [32].

#### 3.2.5 Energy‐dispersive X‐ray spectroscopy

**Fig. 6:**
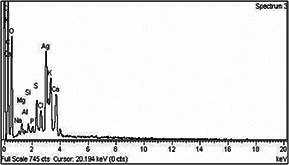
Graphical depiction of EDX values of Q. semecarpifolia synthesised AgNPs

The EDX pattern of synthesised AgNPs revealed strong signs for the silver element (Fig. 6). It is clearly revealed from the EDX profile that the bio‐inspired AgNPs have crystalline morphology and peaks of silver atoms are located in the range of 2–4 keV along with other elements, i.e. carbon, nitrogen, oxygen, aluminium, silicon, sulphur, chlorine, potassium, and calcium [33].

#### 3.2.6 Simultaneous TG‐DTA

**Fig. 7:**
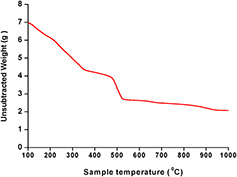
Graphical representation of TGA profile of synthesised AgNPs of Q. semecarpifolia

To study the thermal features of AgNPs, TGA was performed. The TGA is generally used to investigate the change in mass with respect to temperature, which specifies the moisture content, material purity and heat resistance of the AgNPs. Fig. 7 shows the TGA, which clearly figures out that upon increasing temperature weight loss of AgNPs occurs. The sample is heated from 100 to 1000°C for the decomposition of AgNPs*.* The decomposition of the test sample starts at 100°C. Slowly the size decreases when the temperature reached 361.23°C. In the start of the process, the sample size was 6.963 mg but by increasing the temperature, the size of the test sample and its weight was decreased up to 2.711 mg at 526.81°C because the moisture content from AgNPs was removed. After that, no further weight loss occurred [34].

**Fig. 8:**
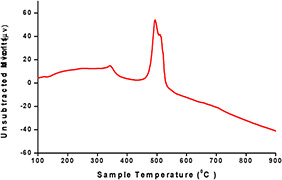
Graphical representation of DTA profile of synthesised AgNPs of Q. semecarpifolia

Another thermal analysis, DTA, is used for the analysis of the change in the temperature of the test sample when subjected to heating. Fig. 8 shows a peak of 492.60°C, and it was concluded that the weight loss was because of the decomposition of organic matter [35].

### 3.3 Biological evaluation of AgNPs

#### 3.3.1 Antioxidant activity

**Fig. 9:**
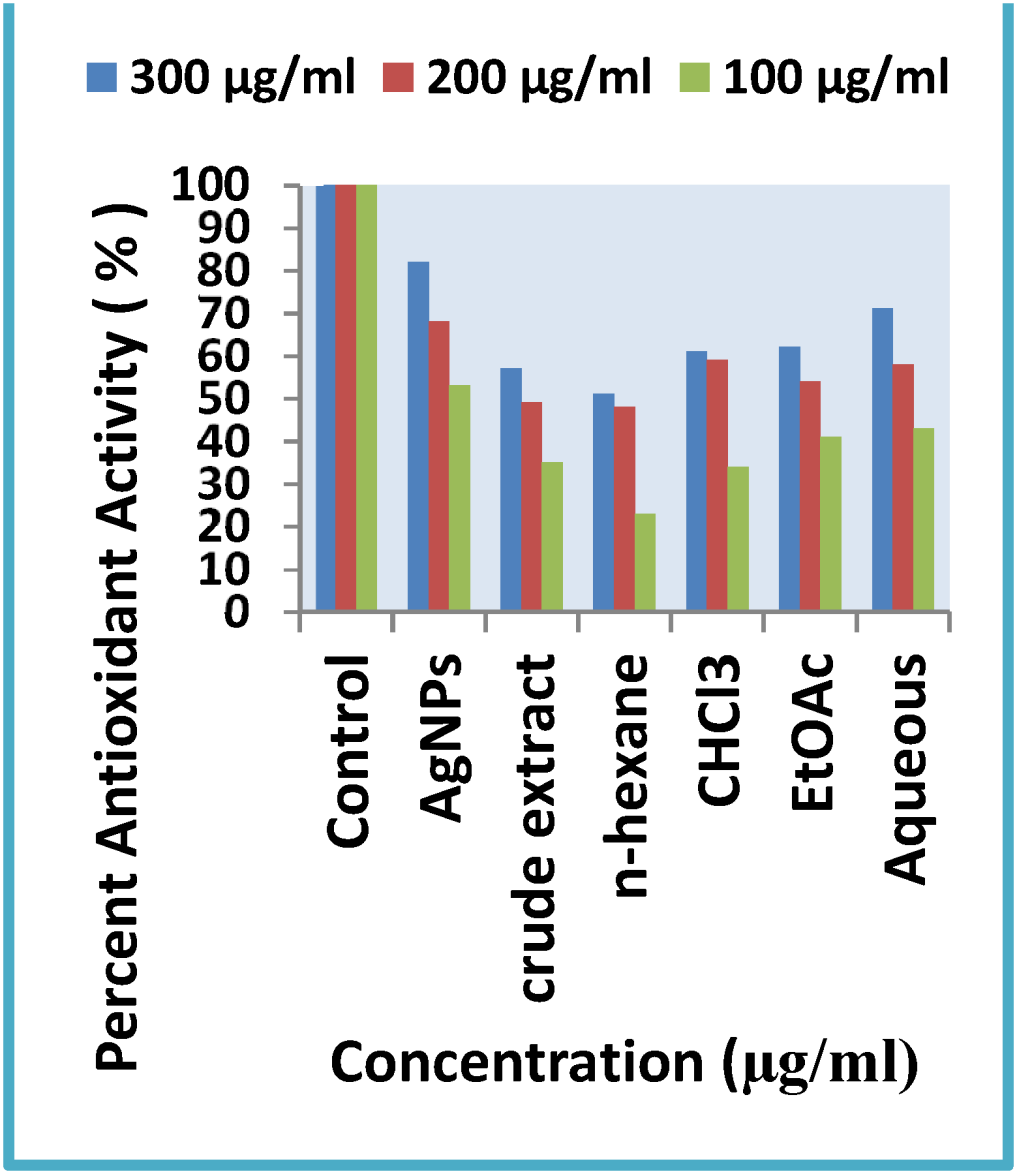
Graphical representation of antioxidant activity by Q. semecarpifolia

Antioxidant activity is because of the redox potential of phytochemicals present in the aqueous leaf extract which could play an important part in quenching singlet and triplet oxygen, decomposing the peroxides or quashing the free radicals [36]. Therefore, it is suggested that the higher antioxidant activity of NPs is due to the adsorption of the antioxidant material from the extract to the surface of the NPs (Fig. 9).

In the antioxidant assay, AgNPs from *Q. semecarpifolia* showed excellent activity (82%) at higher concentration. Similarly, all extracts showed the highest antioxidant activity at 300 μg/ml. The aqueous extract of *Q. semecarpifolia* showed a significant scavenging activity (71%). Moreover, ethyl acetate and chloroform extract showed good activity at 300 µg/ml (62%, 61%), respectively. The crude extract and *n* ‐hexane extract exhibit moderate (57 and 51%) radical scavenging activity, respectively. Therefore, at the highest dilution of 300 µg/ml, the radical scavenging potential of AgNPs and crude extracts considerably increased.

#### 3.3.2 Brine shrimp cytotoxic assay

**Fig. 10:**
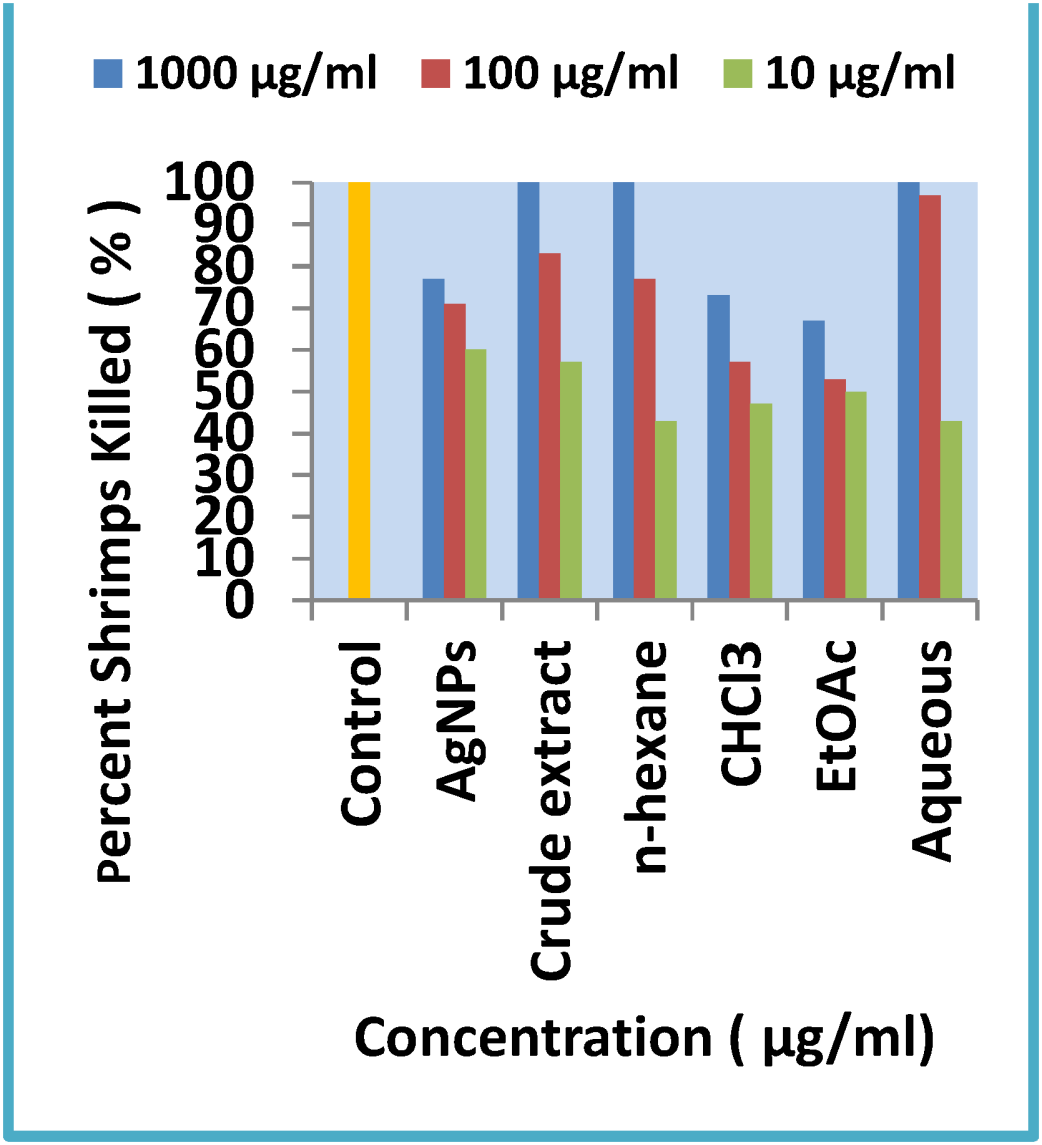
Graphical representation of cytotoxic activity by Q. semecarpifolia

According to the cytotoxic evaluation, it was manifested that crude methanolic, *n* ‐hexane and aqueous fractions of *Q. semecarpifolia* exhibited exemplary cytotoxic potential at the highest concentration of 1000 µg/ml. The LD_50_ values recorded were 16.63 µg/ml for methanolic extract, 4.56 µg/ml for *n* ‐hexane fraction and 11.53 µg/ml for aqueous extract (Fig. 10). While a significant cytotoxic activity was exhibited by green AgNPs (77%), chloroform extract (73%) and ethyl acetate fraction (67%) from the leaves of *Q. semecarpifolia* plant at the highest lethal concentration of 1000 µg/ml. The LD_50_ values recorded were 0.413 µg/ml for AgNPs, 34.73 µg/ml for chloroform extract and 42.56 µg/ml for ethyl acetate fraction. At a lower sample concentration of 100 µg/ml, methanolic, *n* ‐hexane and aqueous leaf extracts demonstrated aced activity (83, 77 and 97%), while biosynthesised AgNPs revealed good lethal potency (71%) and chloroform and ethyl acetate fractions exhibited moderate inhibition (57 and 53%), respectively. At a sample concentration of 10 µg/ml moderate cytotoxicity was manifested by all extracts except for AgNPs (60%).

The extent of Brine shrimp cytotoxicity can be co‐related to its smaller size. Smaller the AgNPs size, stronger is the cytotoxicity [37]. This is because AgNPs size has an effect on their penetration through biological membranes, uptake by cells and immunological reactions initiated against it [38]. The cytotoxicity of NPs towards *A. salina* nauplii maybe in connection with anticancer activity and thereby NPs can be industrialised to anticancer drugs [39].

#### 3.3.3 Phytotoxic activity

**Fig. 11:**
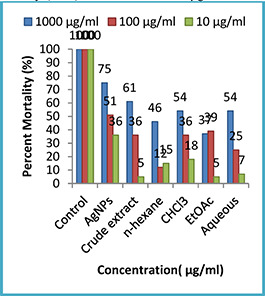
Graphical representation of phytotoxic activity by Q. semecarpifolia

In our study, the phytotoxic effect of various fractions and synthesised AgNPs was tested at different concentrations of 1000, 100 and 10 µg/ml to observe any frond damage (Fig. 11). The phytotoxic assay showed that AgNPs from the leaves of the selected plant possess minimal per cent inhibition at minimal test concentration (10 µg/ml). The per cent inhibition amplifies when the sample concentration is increased three‐fold, i.e. 1000 µg/ml. The results revealed that the AgNPs of *Q. semecarpifolia* exhibited good phytotoxic potential (75%) at a concentration of 1000 μg/ml, moderate (51%) at 100 μg/ml and low activity (36%) was observed at 10 μg/ml.

In contrast to AgNPs, a crude extract, chloroform, and aqueous extract exhibited moderate activity (61, 54 and 54%) at the highest concentration of 1000 µg/ml, respectively. Finally, *n* ‐hexane and ethyl acetate leaf extracts exhibited low activity at all test sample concentrations. Previously, medicinal plants have been investigated for their inhibitory properties against plants. All these investigations have confirmed the existence of variable amounts of phytotoxicity possessed by the plants studied [40, 41].

#### 3.3.4 Haemagglutination activity

Plants and animals contain carbohydrate binding proteins called lectins, which have the unique property to distinguish between different carbohydrate moieties. Studies have been conducted on the structural and functional roles of carbohydrates on the basis of the specificity of lectins [42] (Table 1).

**Table 1:** Tabular representation of haemagglutination activity by *Q. semecarpifolia*

Blood groups	AB−, AB+, O+, O−, A−, A+, B−, B+			
dilutions	1:2	1:4	1:8	1:16
crude extract	—	—	—	—
*n* ‐hexane	—	—	—	—
CHCl_3_	—	—	—	—
EtOAc	—	—	—	—
aqueous	—	—	—	—
AgNPs	—	—	—	—

Lectins can be used as blood typing agents, for the identification of sugar components of the normal and cancerous cells and for the estimation of virus particles [43]. According to the properties of lectins, the haemagglutination activity of *Q. semecarpifolia* was determined against human RBCs. No agglutination of RBCs was seen by the test samples specifying that the plant species lack phytolectins.

## 4 Conclusion

Green biogenic synthesis of NPs has several advantages, for instance, it makes the process easily scalable and economically emergent. A fast, environment‐friendly and useful process for the production of AgNPs using *Q. semecarpifolia* leaf extract has been developed which did not require any chemical substrates. This finding subsequently supported the plant‐mediated process with the advantage of being eco‐friendly. In the present study, the colour of the solution changes during the process by the phytochemicals present in the plant resulting in the formation of AgNPs which is confirmed by the UV–Vis study. Furthermore, from SEM and TEM analysis, it is confirmed that the synthesised NPs are polydispersed and are in round shape having a size ranging from 20 to 50 nm with an average size of 28.40 nm. The XRD study showed that the particles are crystalline in nature. Moreover, the synthesised AgNPs have many biological properties.
